# The Amino Acid Metabolic and Carbohydrate Metabolic Pathway Play Important Roles during Salt-Stress Response in Tomato

**DOI:** 10.3389/fpls.2017.01231

**Published:** 2017-07-17

**Authors:** Zhi Zhang, Cuiyu Mao, Zheng Shi, Xiaohong Kou

**Affiliations:** ^1^School of Food Science and Nutrition Engineering, China Agricultural University Beijing, China; ^2^School of Chemical Engineering and Technology, Tianjin University Tianjin, China

**Keywords:** RNA-seq, tomato, salt stress, amino acid metabolism, carbohydrate metabolism, TOPSIS

## Abstract

Salt stress affects the plant quality, which affects the productivity of plants and the quality of water storage. In a recent study, we conducted the Technique for Order Preference by Similarity to Ideal Solution (TOPSIS) analysis and RNA-Seq, bioinformatics study methods, and detection of the key genes with qRT-PCR. Our findings suggested that the optimum salt treatment conditions are 200 mM and 19d for the identification of salt tolerance in tomato. Based on the RNA-Seq, we found 17 amino acid metabolic and 17 carbohydrate metabolic pathways enriched in the biological metabolism during the response to salt stress in tomato. We found 7 amino acid metabolic and 6 carbohydrate metabolic pathways that were significantly enriched in the adaption to salt stress. Moreover, we screened 17 and 19 key genes in 7 amino acid metabolic and 6 carbohydrate metabolic pathways respectively. We chose some of the key genes for verifying by qRT-PCR. The results showed that the expression of these genes was the same as that of RNA-seq. We found that these significant pathways and vital genes occupy an important roles in a whole process of adaptation to salt stress. These results provide valuable information, improve the ability to resist pressure, and improve the quality of the plant.

## Introduction

Tomato is an important and nutritious vegetable crop worldwide. However, cultivated tomatoes are susceptible under a wide range of environmental pressures (Zhu et al., 2014; Zushi et al., 2014; Sasidharan and Voesenek, 2015). High salinity, for example, can have harmful effects, such as reducing germination, inhibiting growth and reducing fruit production (Xie et al., 2000; Cuartero et al., 2006).

Salt stress can adversely affect the growth and productivity of plants. In the course of growth and development, plants have developed many physiological and biochemical mechanisms to adapt to the stress of the environment (Zhu et al., 2014), and a large number of physiological and metabolic reactions are produced in plants to accommodate this change (Yamaguchi-Shinozaki and Shinozaki, 2006; Hirayama and Shinozaki, 2010; Lata and Prasad, 2011; Tang et al., 2012). By studying the patterns of gene expression in different stress conditions, the adaptive mechanism of different stresses was analyzed (Zhu, 2002).

Salt stress has been observed that amino acids and carbohydrates increase greatly (Fougère et al., 1991). Haitao et al. (2013) demonstrated that arginase expression modulates abiotic stress tolerance in Arabidopsis. It was suggested by Tattini et al. (1996) that in olive leaves, mannitol and glucose play an active role in the osmotic adaptation of plants to salinity. These studies clearly demonstrate that the protein metabolism and carbohydrate metabolism play very important roles in the stress response. We can more fully understand gene function by studying the plant transcriptome. The RNA sequence is a new, efficient and rapid way to conduct transcriptional research and explore the mechanisms that exist in cells (Gal, 1980; Sultan et al., 2008; Trapnell et al., 2010). Despite progress in understanding how protein metabolism and carbohydrate metabolism participate in the adaptation to salt stress, the key genes and signaling pathways involved in these processes remain unclear.

Therefore, we use the RNA-seq screen for obvious pathways and key genes and protein metabolism and carbohydrate metabolism based on the Web gene ontology (GO) (http://wego.genomics.org.cn/cgi-bin/wego/index.pl) and Kyoto Encyclopedia of Genes and Genomes (KEGG) pathway enrichment analysis (http://www.funnet.ws/). TOPSIS (the Technique for Order Preference by Similarity to Ideal Solution) also test and determine the salt concentration and processing time, to provide a base for other researchers. Our aim is to explore the mechanism of protein metabolism regulation and carbohydrate metabolism regulation during the salt stress response in tomato and to provide valuable information to enhance the ability to resist pressure, and improve the quality of the plant. The results of the study can also be used as a reference for the selection of candidate genes in tomato for further functional characterization.

## Materials and methods

### Plant materials and growth conditions

Tomato (*Solanum lycopersicum*) seeds included the Micro-Tom cultivar. A total of 120 seeds of uniform size were surface-sterilized in 70% (v/v) ethanol for 15 s, followed by 4% (w/v) sodium hypochlorite for 15 min, and then use sterile distilled water rinse several times (Cavalcanti et al., 2004). The seeds were germinated at 24°C on wet filter paper and under darkness. When a 0.3 cm-radicle protrusion appeared, the seeds were laid on an agar-solidified MS medium (Murashige and Skoog, 1962) in triplicate, without or supplemented with 50–250 mM sodium chloride (NaCl). They were then moved to an incubator at 24°C in a dark 16 h/light 8 h system. Additionally, the plant height, leaf blade number and antioxidant enzyme activities were measured and analyzed after 7 d, 11 d, 15 d, 19 d, and 23 d treatment. The POD, SOD, and CAT activities were determined according to previous work (Cavalcanti et al., 2004).

### Comprehensive evaluation of salt treatment conditions

The Technique for Order Preference by Similarity to Ideal Solution (TOPSIS) was provided by Hwang and Yoon (1981). The detailed steps are listed below:

Step 1: Create a ranking decision matrix:

$$\begin{array}{l} X_{ij}=\left(\begin{array}{cccc} x_{11} & x_{12} & \cdots & x_{1n}\\ x_{21} & x_{22} & \cdots & x_{2n}\\ \vdots & \vdots & \vdots & \vdots \\ x_{m1} & x_{m2} & \cdots & x_{mn} \end{array}\right),i=1,2,...,m;\;j=1,2,...n \end{array}$$

where *x*_*ij*_ is the rating of alternative *A*_*i*_ (m) with respect to the criterion *C*_*j*_(n) evaluated.

Step2: Calculate the normalized decision matrix *r*_*ij*_:

$$\begin{array}{l} r_{ij}=\frac{x_{ij}}{\sqrt{\sum_{i=1}^{m}xij^{2}}},i=1,2,\ldots ,m;j=1,2,\ldots ,n \end{array}$$

Step 3: A weighted standardized decision matrix is constructed by multiplying the standardized decision matrix times the associated weights. The weighted standardized value *vij* is calculated in the following way:

$$\begin{array}{l} vij=wi\times rij,\sum_{i=1}^{m}wi=1 \end{array}$$

Where *wi* is the weight of the jth criterion.

Step 4: Identify positive and negative ideal solutions:

$$\begin{array}{l} A^{+}=\left\{{v_{1}}^{+},{v_{2}}^{+},\cdots ,{v_{n}}^{+}\right\}\\ =\;\left\{\left(\max_{j}v_{ij}|i\in I\right),\left(\min_{j}v_{ij}|i\in J\right)\right\}\\ A^{-}=\left\{{v_{1}}^{-},{v_{2}}^{-},\cdots ,{v_{n}}^{-}\right\}\\ =\;\left\{\left(\min_{j}v_{ij}|i\in I\right),\left(\max_{j}v_{ij}|i\in J\right)\right\} \end{array}$$

Where I is associated with the benefit criteria and J is associated with the cost criteria.

Step 5: Use Measure two Euclidean distances for both the positive and the negative.

$$\begin{array}{l} {d_{j}}^{+}=\sqrt{\sum_{i=1}^{n}{\left(v_{ij}-{v_{i}}^{+}\right)}^{2};}\;{d_{j}}^{-}=\sqrt{\sum_{i=1}^{n}{\left(v_{ij}-{v_{i}}^{-}\right)}^{2}} \end{array}$$

where *d*_*j*_^+^ and *d*_*j*_^−^ represent the distance from Aj which is expressed separately from the ideal solution of positive and negative.

Step 6: Calculate the relative closeness of the ideal solution and compare the *Rj* value to the alternatives.

$$\begin{array}{l} Rj=dj^{-}/\left(dj^{+}+dj^{-}\right),Rj\in \left[0,1\right] \end{array}$$

where *Rj* represents the relative closeness.

All treatments have three replication operations presented as the mean ± the standard deviation of each experiment. The correlations were estimated using Pearson's correlation coefficient in the IBM SPSS Statistics 20 (SPSS, Chicago, Illinois, USA) software package. We chose the tomato material to be used for RNA-Seq according to the results of the correlation analysis and the TOPSIS analysis.

### RNA-seq analysis

For building the RNA library based on mRNA-seq Illumina company, the tomatos were sequenced using HiSeq 2000 from Shanghai OE Biotech. Co., Ltd. Each sample use 50 ng. The results were compared with the database, each of which was anotated for a follow-up experiment. The standard for RNA is RIN ≥ 7, 28S/18S>0.7. Both the control and salt treated samples had three repetitions, respectively.

### Quantitative real-time PCR (qRT-PCR) verification

Extract the total RNA use the RNeasy extraction tool. Then reverse-transcribed it into cDNA for the next analysis. Based on the equipment instructions, we used the SYBR fluorescent reagent and the 7900 system to conduct real-time PCR, and analyzed data using Microsoft Excel with the 2^−ΔΔCt^ relative quantitative method.

## Results

### Comprehensive evaluations of the salt concentration and treatment time

Based on a correlation analysis (Table 1), all of the indicators are strongly correlated with the salt concentration, suggesting effective indicators that represent the salt tolerance of tomato. The TOPSIS analysis revealed the rank of *R*_*j*_ considering all indicator values for different salt concentrations during the response to salt stress (Table 2); 200 mM was the optimum NaCl concentration for the identification of salt tolerance. Furthermore, the TOPSIS analysis examined the rank of *R*_*j*_ considering of the indicator values for different salt treatment times under the 200 mM NaCl treatment (Table 3) and indicated that 19 d is the optimum salt treatment time for the identification of salt tolerance. Accordingly, tomatoes treated without or with 200 mM NaCl for 19 d were chosen for further RNA-Seq analysis.

**Table 1:** Correlation analysis in seedling stage.

**Indicator**	**Correlation coefficient**	**Indicator**	**Correlation coefficient**
7d plant height	−0.957^**^	15d SOD activity	0.909^*^
7d leaf blade number	−0.960^**^	15d CAT activity	0.65
7d POD activity	0.833^*^	19d plant height	−0.944^**^
7d SOD activity	0.960^**^	19d leaf blade number	−0.970^**^
7d CAT activity	0.916^*^	19d POD activity	0.898^*^
11d plant height	−0.938^**^	19d SOD activity	0.961^**^
11d leaf blade number	−0.938^**^	19d CAT activity	0.824^*^
11d POD activity	0.793	23d plant height	−0.989^**^
11d SOD activity	0.904^*^	23d leaf blade number	−0.931^**^
11d CAT activity	0.924^**^	23d POD activity	0.929^**^
15d plant height	−0.922^**^	23d SOD activity	0.922^**^
15d leaf blade number	−0.985^**^	23d CAT activity	0.854^*^
15d POD activity	0.846^*^		

Single (^*^P < 0.05) and double (^**^P < 0.01) asterisks denote statistically significant correlation between indicators under different treatments. The minus sign represents the negative correlation.

**Table 2:** Appropriate treatment concentration evaluated by the TOPSIS method.

**Concentration**	***d_j_^+^***	***d_j_^−^***	***R*_*j*_**	**Rank**
0 mM	1.8515	0	0	6
50 mM	1.4561	0.3166	0.1786	5
100 mM	1.0262	0.6325	0.3813	4
150 mM	0.9217	0.7883	0.461	3
200 mM	0.2262	1.4237	0.8629	1
250 mM	0.3819	1.3864	0.784	2

*${d_{j}}^{+}$, Positive ideal solution of Euclidean distance; ${d_{j}}^{-}$, Negative ideal solution of Euclidean distance; R_j_, The closeness coefficient*.

**Table 3:** Appropriate treatment time evaluated by the TOPSIS method.

**Time**	***d_j_^+^***	***d_j_^−^***	***R*_*j*_**	**Rank**
7d	1.0664	0	0	5
11d	0.6952	0.1435	0.1711	4
15d	0.3626	0.4338	0.5447	2
19d	0.0985	0.741	0.8827	1
23d	0.4511	0.4728	0.5117	3

*${d_{j}}^{+}$, Positive ideal solution of Euclidean distance; ${d_{j}}^{-}$, Negative ideal solution of Euclidean distance; R_j_, The closeness coefficient*.

### The differential expression analysis and go classification of expressed genes

**Figure 1:**
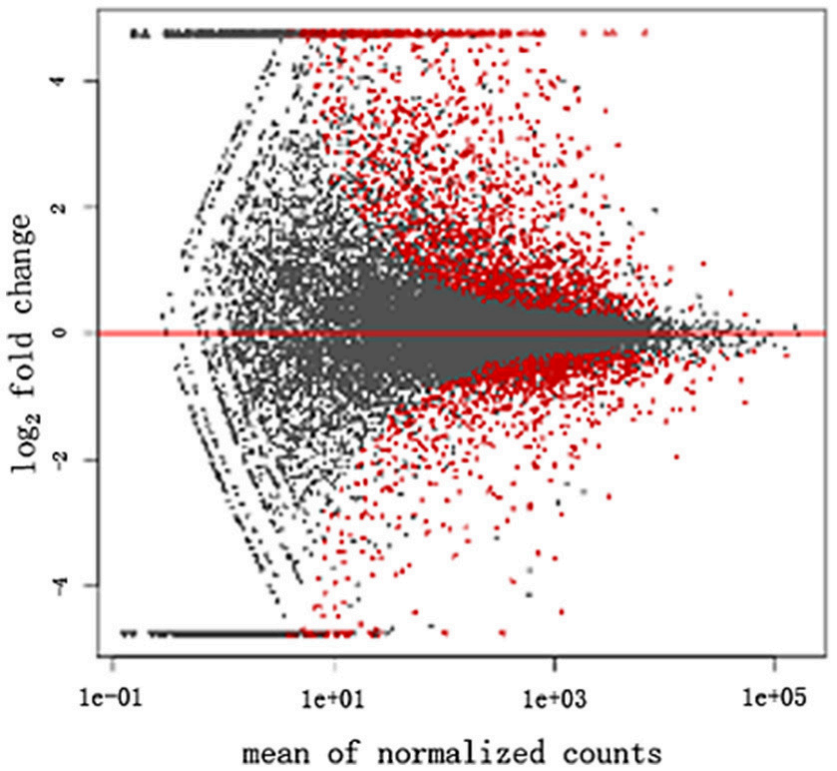
Volcano plot of the differentially displayed genes in tomato.

**Figure 2:**
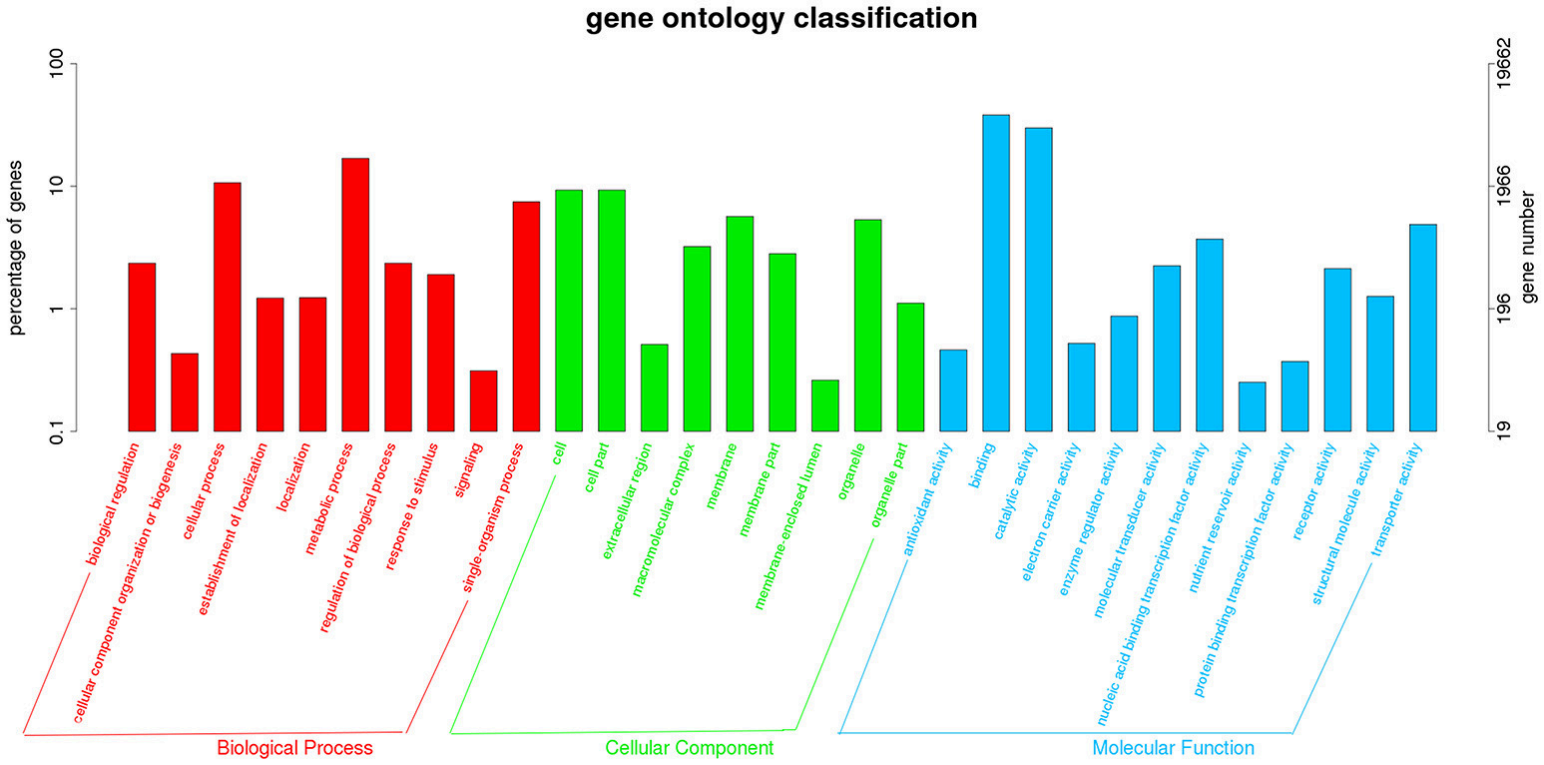
Histogram representation of Gene Ontology classification.

The transcriptome data were analyzed with RNA-Seq technology. Based on RNA-seq, we did the variance analysis and the differential expression gene was selected according to the standard of *P* < 0.05. The false discovery rate (FDR) was set to 0.001 to determine the threshold of the *P*-value for multiple tests. The absolute value of |log_2_Ratio| ≥ 1 was used to determine the difference between the gene expression transcription group and the database. The 3,792 different genes were discovered during the salt stress response, including 1,498 up-regulated genes and 2,294 down-regulated genes (Figure 1). Gene function, annotation, and classification were researched by GO analysis. The 2,776 genes were founded to participated in the metabolic processes (Figure 2).

### Pathway enrichment analysis of differentially displayed genes in the amino acid metabolic pathway and carbohydrate metabolic pathway

**Figure 3:**
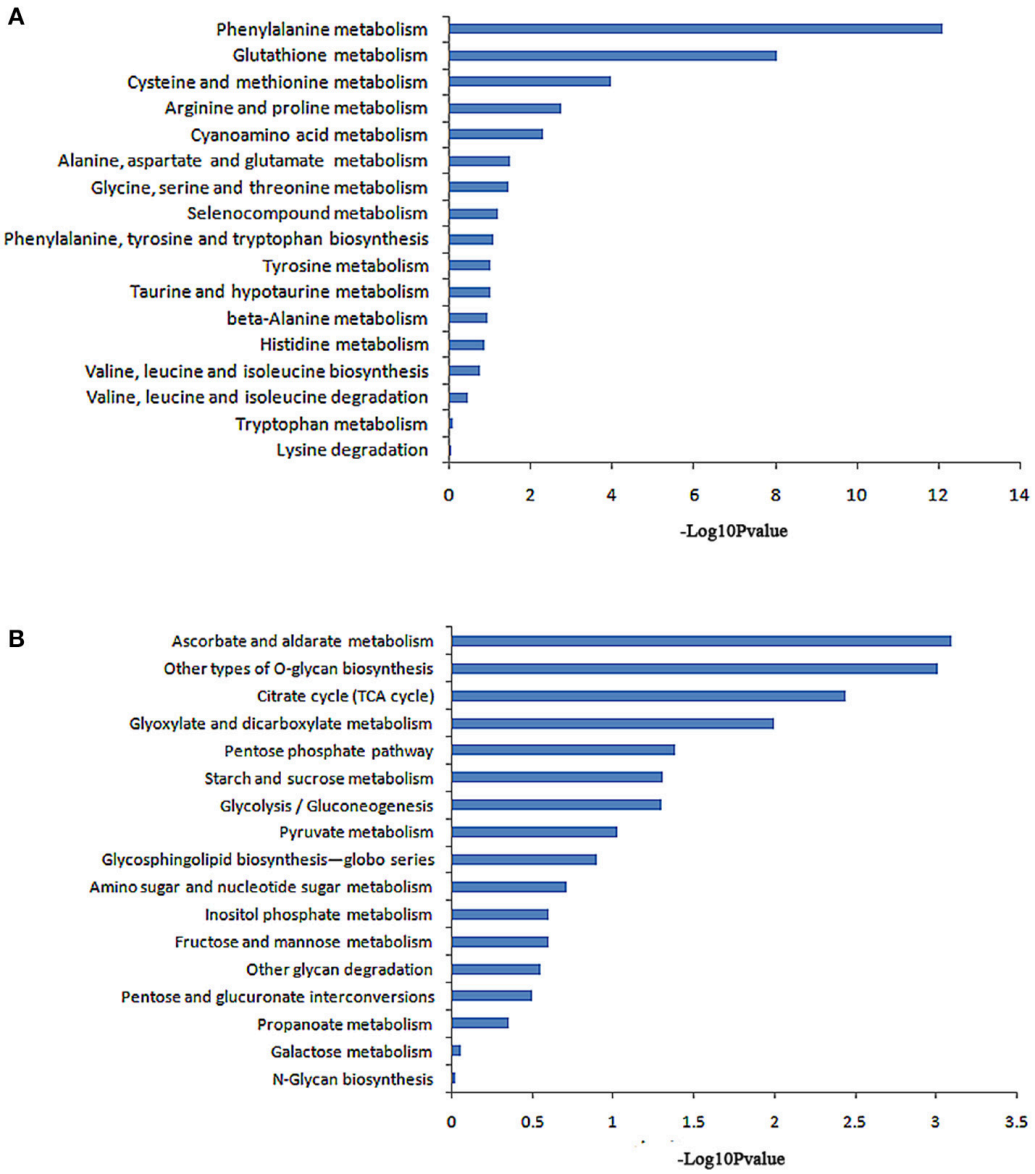
Pathway enrichment analysis of differentially displayed genes. **(A)** Amino acid metabolic pathway, **(B)** Carbohydrate metabolic pathway.

The KEGG database analysis revealed 45 biological metabolic pathways involved in the response to salt stress, including 17 essential amino acid metabolic pathways. A total of 262 genes that participated in amino acid metabolism were found to be enriched in the transcriptome. In these amino acid metabolic pathways, there are seven distinct pathways (*P* < 0.05): phenylalanine metabolism; glutathione metabolism; cysteine and methionine metabolism; arginine and proline metabolism; cyanoamino acid metabolism; alanine, aspartate and glutamate metabolism; and glycine, serine and threonine metabolism. The remaining 10 metabolic pathways exhibited no significant difference (Table 4, Figure 3A).

**Table 4:** Amino acid metabolic pathway enrichment analysis of differentially displayed genes.

**Pathway**	**Gene numbers**	***P*-value**	**Pathway ID**
Phenylalanine metabolism	62	8.15E-13	PATH:sly00360
Glutathione metabolism	40	9.73E-09	PATH:sly00480
Cysteine and methionine metabolism	29	0.00011022	PATH:sly00270
Arginine and proline metabolism	23	0.001806064	PATH:sly00330
Cyanoamino acid metabolism	13	0.004924912	PATH:sly00460
Alanine, aspartate and glutamate metabolism	12	0.033982915	PATH:sly00250
Glycine, serine and threonine metabolism	15	0.037152102	PATH:sly00260
Selenocompound metabolism	4	0.067923439	PATH:sly00450
Phenylalanine, tyrosine and tryptophan biosynthesis	11	0.087873895	PATH:sly00400
Tyrosine metabolism	9	0.102603106	PATH:sly00350
Taurine and hypotaurine metabolism	3	0.104481727	PATH:sly00430
beta-Alanine metabolism	11	0.120123385	PATH:sly00410
Histidine metabolism	8	0.139929408	PATH:sly00340
Valine, leucine and isoleucine biosynthesis	5	0.182742699	PATH:sly00290
Valine, leucine and isoleucine degradation	11	0.356166502	PATH:sly00280
Tryptophan metabolism	3	0.86107794	PATH:sly00380
Lysine degradation	3	0.962424599	PATH:sly00310

A total of 231 genes that participated in carbohydrate metabolism were found to be enriched in the transcriptome. KEGG database analysis revealed 17 carbohydrate metabolism pathways. In these pathways, six greatly different pathways were detected (*P* < 0.05): an ascorbate and aldarate metabolic pathway; other types of O-glycan biosynthesis; a citrate cycle (TCA cycle); glyoxylate and dicarboxylate metabolism; a pentose phosphate pathway; and starch and sucrose metabolism. The remaining 11 metabolic pathways showed no significant difference (Table 5, Figure 3B).

**Table 5:** Carbohydrate metabolic pathway enrichment analysis of differentially displayed genes.

**Pathway**	**Gene numbers**	***P*-value**	**Pathway ID**
Ascorbate and aldarate metabolism	16	0.000811885	PATH:sly00053
Other types of O-glycan biosynthesis	4	0.000994137	PATH:sly00514
Citrate cycle (TCA cycle)	16	0.003646264	PATH:sly00020
Glyoxylate and dicarboxylate metabolism	16	0.010261383	PATH:sly00630
Pentose phosphate pathway	13	0.042122932	PATH:sly00030
Starch and sucrose metabolism	42	0.049607427	PATH:sly00500
Glycolysis/Gluconeogenesis	25	0.051214561	PATH:sly00010
Pyruvate metabolism	19	0.095644452	PATH:sly00620
Glycosphingolipid biosynthesis-globo series	2	0.127871107	PATH:sly00603
Amino sugar and nucleotide sugar metabolism	22	0.197655745	PATH:sly00520
Inositol phosphate metabolism	13	0.254546349	PATH:sly00562
Fructose and mannose metabolism	10	0.255377989	PATH:sly00051
Other glycan degradation	2	0.284825376	PATH:sly00511
Pentose and glucuronateinterconversions	18	0.320047239	PATH:sly00040
Propanoate metabolism	6	0.450774927	PATH:sly00640
Galactose metabolism	5	0.890373237	PATH:sly00052
N-Glycan biosynthesis	2	0.966898356	PATH:sly00510

### Screening of key regulatory genes participating in amino acid metabolism and carbohydrate metabolism during the response to salt stress

**Figure 4:**
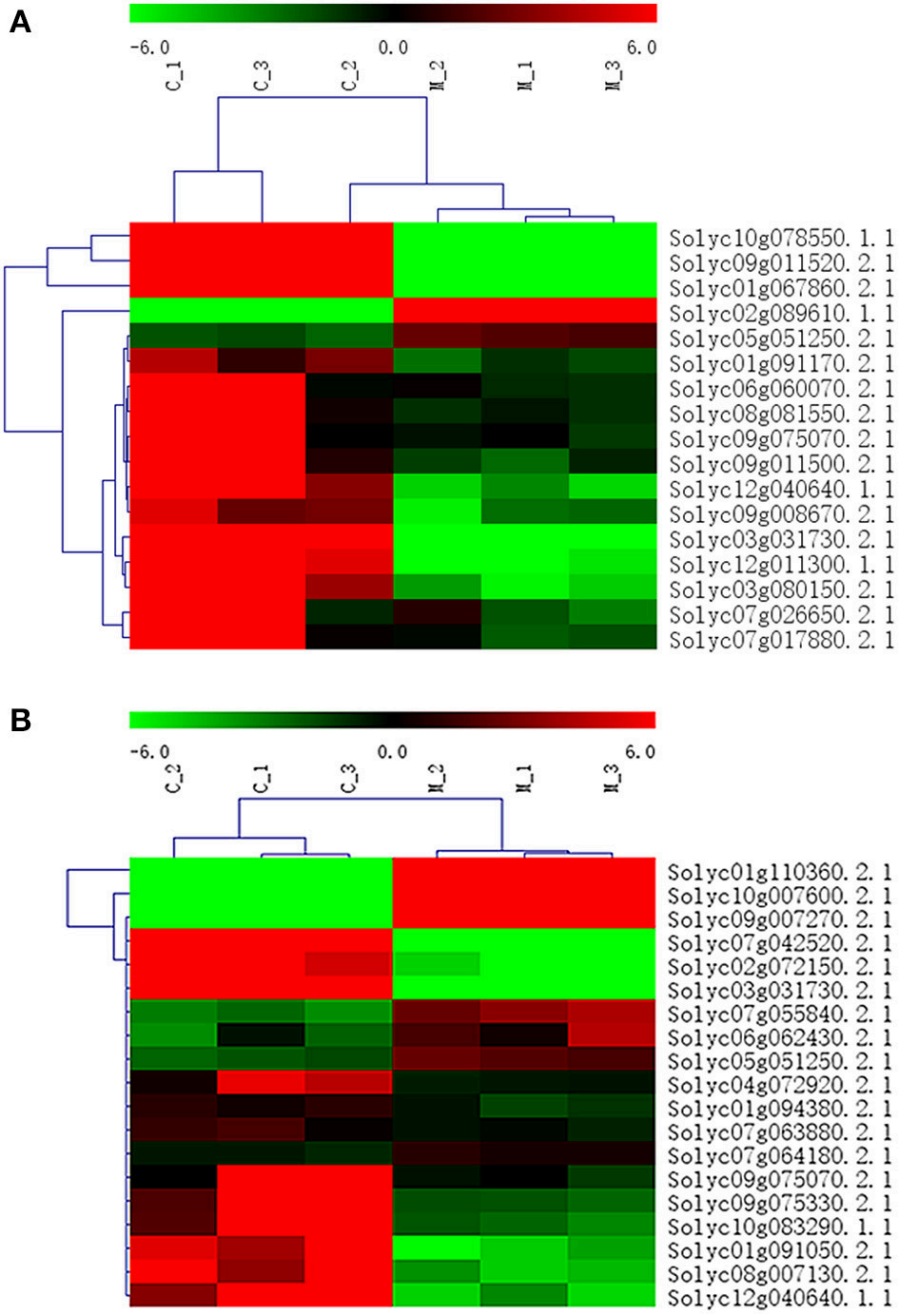
Heat map representation of differentially displayed genes. **(A)** Amino acid metabolic pathway, **(B)** Carbohydrate metabolic pathway.

In addition to *P* < 0.05, we further screened differentially expressed genes with the two new standards to ensure the availability of screening results: fragments per kb per million reads (fpkm)>0.05 and |log_2_Fold Change| ≥ 1. Based on these standards, we screened 17 genes in seven amino acid metabolic pathways (Table 6) and 19 genes in six carbohydrate metabolic pathways (Table 7). To forecast the expression profiles of the genes during the tomato salt tolerance process, we conducted a cluster analysis using a heat map (Figure 4). As shown in Figure 4A, Solyc05g051250.2.1 and Solyc02g089610.1.1 exhibited an increase in expression during the response to salt stress. The opposite expression patterns were observed in the15 other genes in the amino acid metabolic pathway. As shown in Figure 4B, Solyc06g062430.2.1, Solyc09g007270.2.1, Solyc07g055840.2.1, Solyc05g051250.2.1, Solyc10g007600.2.1, Solyc01g110360.2.1, and Solyc07g064180.2.1 exhibited an increase in expression during the salt stress response. Opposite expression patterns were observed for 12 other genes in the carbohydrate metabolic pathway.

**Table 6:** Related regulatory genes in amino acid metabolic pathway.

**KEGG pathway**	**Gene ID**	**Gene name**
Alanine, aspartate and glutamate metabolism	Solyc05g051250.2.1	LOC101261030
	Solyc10g078550.1.1	gdh1
Arginine and proline metabolism	Solyc01g091170.2.1	ARG2
	Solyc02g089610.1.1	LOC101260400
Cyanoamino acid metabolism	Solyc03g031730.2.1	LOC101255272
	Solyc09g075070.2.1	LOC101248047
	Solyc12g040640.1.1	LOC101246223
Cysteine and methionine metabolism	Solyc06g060070.2.1	LOC101266529
	Solyc07g026650.2.1	ACO5
	Solyc08g081550.2.1	LOC101258353
Glutathione metabolism	Solyc09g011500.2.1	LOC101268216
	Solyc09g011520.2.1	LOC101267638
	Solyc12g011300.1.1	LOC101265897
Glycine,serine and threonine metabolism	Solyc09g008670.2.1	LOC543983
Phenylalanine metabolism	Solyc01g067860.2.1	LOC101247458
	Solyc03g080150.2.1	LOC101252368
	Solyc07g017880.2.1	LOC101264739

**Table 7:** Related regulatory genes in carbohydrate metabolic pathway.

**KEGG pathway**	**Gene ID**	**Gene name**
Ascorbate and alarate metabolism	Solyc06g062430.2.1	LOC101263222
	Solyc09g007270.2.1	LOC101258987
Citrate cycle (TCA cycle)	Solyc07g055840.2.1	LOC101258079
Glyoxylate and dicarboxylate metabolism	Solyc05g051250.2.1	LOC101261030
	Solyc10g007600.2.1	LOC100134875
Other types of O-glycan biosynthesis	Solyc01g094380.2.1	LOC101261174
Pentose phosphate pathway	Solyc01g110360.2.1	LOC101246870
Starch and sucrose metabolism	Solyc01g091050.2.1	LOC101247960
	Solyc02g072150.2.1	LOC101245612
	Solyc03g031730.2.1	LOC101255272
	Solyc04g072920.2.1	LOC101249633
	Solyc07g042520.2.1	LOC101267720
	Solyc07g063880.2.1	LOC101262329
	Solyc07g064180.2.1	PME2.1
	Solyc08g007130.2.1	LOC101259175
	Solyc09g075070.2.1	LOC101248047
	Solyc09g075330.2.1	LOC101266973
	Solyc10g083290.1.1	Wiv-1
	Solyc12g040640.1.1	LOC101246223

### qRT-PCR verification

**Figure 5:**
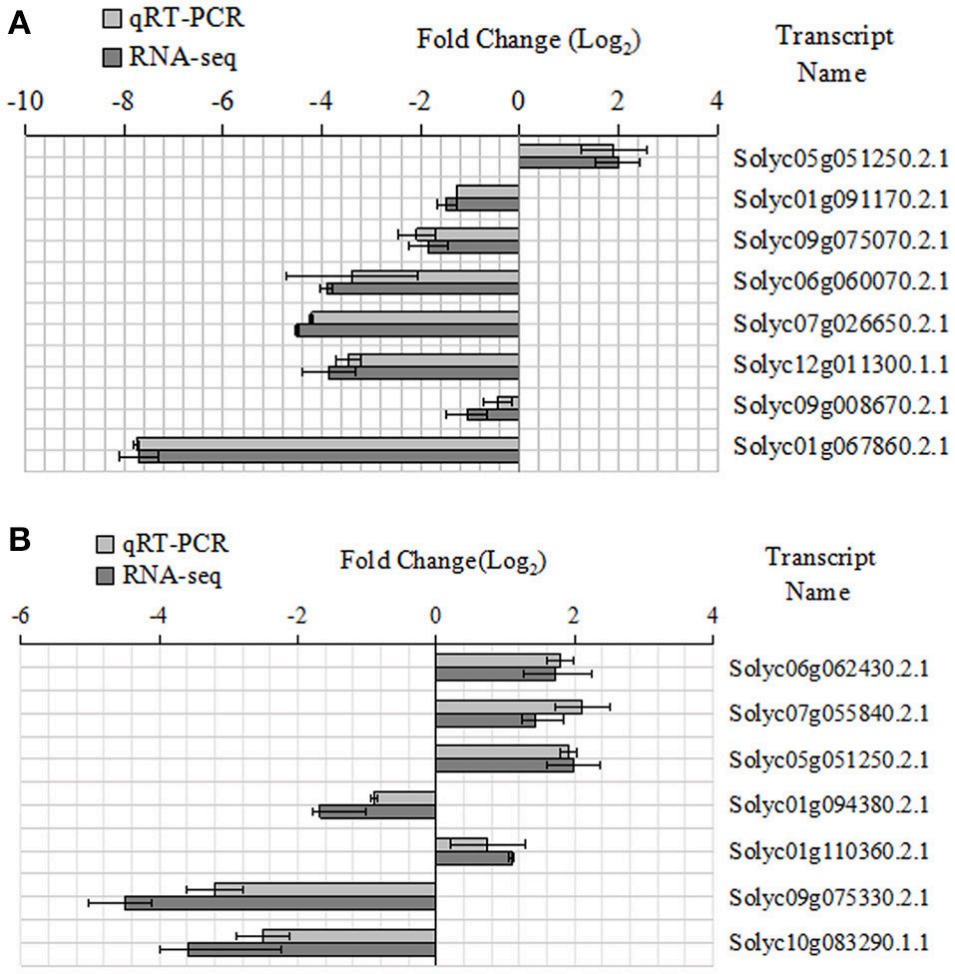
Expression patterns of the different genes between the qRT-PCR and RNA-seq. **(A)** Amino acid metabolic pathway, **(B)** Carbohydrate metabolic pathway.

Some key genes were chosen in Tables 6, 7. We analyed their expression levels in tomatoes treated without NaCl as a control and in tomatoes treated with 20 mM NaCl (Figure 5). According to the results, Solyc05g051250.2.1 was up-regulated, which is involved in Alanine, aspartate and glutamate metabolism, by contrast, Solyc01g091170.2.1 (ARG2) involved in Arginine and proline metabolism, Solyc09g075070.2.1 involved in Cyanoamino acid metabolism, Solyc06g060070.2.1 and Solyc07g026650.2.1 (ACO5) involved in Cysteine and methionine metabolism, Solyc12g011300.1.1 involved in Glutathione metabolism, Solyc09g008670.2.1 involved in Glycine,serine and threonine metabolism and Solyc01g067860.2.1 involved in Phenylalanine metabolism were down-regulated (Figure 5A). Meanwhile, Solyc06g062430.2.1 involved in Ascorbate and alarate metabolism, Solyc07g055840.2.1 involved in Citrate cycle (TCA cycle), Solyc05g051250.2.1 involved in Glyoxylate and dicarboxylate metabolism and Solyc01g110360.2.1 involved in Pentose phosphate pathway were up-regulated, by contrast, Solyc01g094380.2.1 involved in Other types of O-glycan biosynthesis, Solyc09g075330.2.1 and Solyc10g083290.1.1 (Wiv-1) involved in Starch and sucrose metabolism were down-regulated (Figure 5B). The expression of these genes is mainly the same as the RNA-seq results.

## Discussion

The adaptation of plant cells in salt stress is closely relative to various metabolic processes. It has been suggested that proline metabolism (Khedr et al., 2003), ascorbic acid (ASA) metabolism (Zushi et al., 2014), and other pathways play important roles during the salt stress response. In these metabolic processes, amino acid and carbohydrates are the main players in various metabolic and regulatory pathways. They are responsible for biological cell adaptation. Therefore, amino acid metabolism and carbohydrate metabolism are crucial to the salt stress response. In our study, we found that in the adaptation process, the differentially expressed genes were significantly enriched in 7 amino acid metabolic pathways and in 6 carbohydrate metabolic pathways.

Many studies have found that amino acid metabolism is closely related to abiotic stress tolerance (Fougère et al., 1991; Zörb et al., 2004; Haitao et al., 2013). We identified 7 significant amino acid metabolic pathways and verified 8 key genes. In our study, the ARG2 gene encoding arginase 2 (Solyc01g091170.2.1), involved in arginine and proline metabolism, was significantly enriched. Arginine is an vital amino acid to transport and storage the nitrogen and occupy a precursor to synthesis other amino acids or polyamines (Flores et al., 2008; Brauc et al., 2012). The arginase 2 genes variations associate with steroid response, which probably plays an important role in asthma development, severity and progression (Vonk et al., 2010). The AtARG2-knockout Arabidopsis could enhance environmental stress tolerance compared with the wild type, and increased tolerance was suggested by changes in physiological parameters, containing electrolyte leakage, water loss, porosity and surial rate (Haitao et al., 2013). However, in the past, most attention has focused on the function of proline which occupy a compatible osmolyte (Yancey et al., 1982) and osmoprotectant (Serrano and Gaxiola, 1994), It is much less concerned with the further role of stress tolerance. Our study suggested that proline metabolic pathways exhibit significant differences during the salt stress response (*P* < 0.05). Consistently, Khedr et al. (2003) reported that proline induces the expression of salt-stress-responsive proteins, which may increase the adaptation of *Pancratium maritimum* L. to response salt stress. The gene encoding beta-glucosidase 11-like (Solyc09g075070.2.1) was down-regulated in our study, which is involved in Cyanoamino acid metabolism. The role of beta-glucosidase is the hydrolysis of terminal, non-reducing beta-D-glucose residues with the increase of beta-D-glucose. Compared to the control group, the enzyme increased in the corn roots and buds (Zörb et al., 2004). Structure of acid beta-glucosidase with pharmacological chaperone provides insight into Gaucher disease (Raquel et al., 2007). López-Berenguer et al. (2008) demonstrated that cysteine and methionine decreased significantly in broccoli after salt application. Furthermore, the expression of the ACO5 gene encoding the 1-aminocyclopropane-1-carboxylate oxidase (Solyc07g026650.2.1), which is involved in cysteine and methionine metabolism, was observed to be down-regulated throughout the salt stress response. An increase in ethylene biosynthesis of arabidopsis is releated to the transcript of ACO5 under waterlogging stress (López-Berenguer et al., 2008; Sasidharan and Voesenek, 2015). ACO5 osmoregulation in cotton could probably response to water stress (Tschaplinski and Blake, 1989). ACO5 is the specific target gene of the NAC (for no apical meristem [NAM], Arabidopsis transcription activation factor [ATAF], and cup-shaped cotyledon [CUC2]) transcription factors), and many studies have found that NAC is involved in the plant abiotic stressresponse (Xie et al., 2000; Nuruzzaman et al., 2010; Rauf et al., 2013). Hernández et al. (2000) found that the activity of glutathione reductase (GR) accumulated in the salt-tolerant pea compared with the salt-sensitive cultivar after long-term NaCl treatment, indicating that glutathione metabolism is participated in the adaption to salt stress. In our study, the gene encoding a probable glutathione S-transferase (Solyc12g011300.1.1) involved in glutathione metabolism was significantly down-regulated. Cho et al. (2001) reported that Glutathione S-Transferase Mu modulates the stress-activated signals by suppressing apoptosis signal-regulating kinase 1. Roxas et al. (1997) found that hyperexpression glutathione s-transferase enhanced the growth of genetically modified tobacco seedlings suffering stress. Moreover, the gene encoding peroxidase 24 (Solyc01g067860.2.1), which is involved in phenylalanine metabolism, was observed to be significantly down-regulated. Huang et al. (2009) reported that a rice peroxidase 24 precursor was down-regulated and could exhibit peroxidase activity ofscavenging H_2_O_2_ under salt treatment. The total peroxidase activity improved under salt stress, and elevation activity depend on NaCl concentration (Sreenivasulu et al., 1999). SE-glutathione peroxidase play the important role for cell survival against oxidative stress (Carine et al., 1994).

Many studies have shown that carbohydrate metabolism occupies a vital function in abiotic stress tolerance (Fougère et al., 1991; Zörb et al., 2004; Haitao et al., 2013). We identified 6 significant carbohydrate metabolic pathways and verified 7 key genes. Downton (1977) found that NaCl-stressed grapevine leaves included decreased sucrose and starch but improved the reducing sugars levels. In this study, Wiv-1 (Solyc10g083290.1.1), encoding the acid invertase involved in starch and sucrose metabolism, was down-regulated during the response to salt stress. Consistently, Dubey and Singh (1999) reported that acid invertase activity decreased on the shoots of NaCl-tolerant rice but increased on NaCl-sensitive rice. It is suggested that the increasion of sugars and other compatible solutes, contributes to osmotic adjustment under salt stress. In addition, Kim et al. (2000) reported that a maize vacuolar invertase is induced by water stress. In our study, the gene encoding fructose-bisphosphate aldolase 1 (Solyc01g110360.2.1), which is participated in the pentose phosphate pathway, was significantly up-regulated. Chaves et al. (2009) also demonstrated that fructose-bisphosphate aldolase was differently affected by salt stress. Lu et al. (2012) reported that fructose 1,6-bisphosphate aldolase genes in Arabidopsis response to abiotic stresses. The gene encoding citrate synthase 3 (Solyc07g055840.2.1) was up-regulated in our study, which is participated in the Citrate cycle (TCA cycle) pathway. Citrate synthase activity is an indicator of metabolic potential in mitochondria and should increase if there is a net increase in the volume of mitochondria in the tissue. However, there was no difference in citrate synthase activity among freshwater- and seawater-acclimated fish (Marshall et al., 1999). The gene encoding myo-inositol oxygenase 1 (Solyc06g062430.2.1) was up-regulated in our study, which is participated in the Ascorbate and alarate metabolism. The vital function of myo-inositol is showed to be relative to osmotic balance and transport the Na + from roots to shoots (Nelson and Bohnert, 1999). The activity of inositol and inositol oxygenase has been found to catalyze the oxidation of the d-glucuronate free inositol (Lorence et al., 2004). Cotsaftis et al. (2011) reported that myo-inositol oxygenase was down-regulated in salt-tolerant rice.

The gene encoding glutamine synthetase-like (Solyc05g051250.2.1) was up-regulated in our study and is involved in both amino acid and carbohydrate metabolism. Glutamine synthetase is beneficial to nitrogen assimilation and has been founded in prokaryotes and eukaryotes (Doskočilová et al., 2011). Viégas and Silveira (1999) found that salinity imposed sensitivity on nitrogen assimilation. The higher the sensitivity imposed, the more severe the salt-injurious affects on plant development. Meanwhile, ${\text{NO}}_{3}^{-}$ uptake and its assimilation were suggested to limit the nitrogen assimilation under salt stress (Silveira et al., 2001). Furthermore, genes involved in starch and sucrose metabolism (Solyc09g075330.2.1), cysteine and methionine metabolism (Solyc06g060070.2.1), glycine, serine and threonine metabolism (Solyc09g008670.2.1) and other types of o-glycan biosynthesis (Solyc01g094380.2.1) were observed to exhibit significant expression after salt stress, indicating that they were involved in the response to salt stress, but their impact on stress adaptation has not yet been reported. Therefore, these specific regulatory mechanisms need to be explored in future.

In conclusion, 200 mM and 19d are the optimum salt treatment conditions for the identification of salt tolerance in tomato. Based on RNA-Seq, we analyzed the metabolic pathways and identified some of the relevant critical genes participating in amino acid metabolism and carbohydrate metabolism during the response to salt stress in tomato. These metabolic pathways and key genes play vital roles to response salt stress, which provides valuable information to enhance the ability to resist pressure, improve the quality of the plant and lay a solid foundation for future research.

## Author contributions

XK planned the research. ZZ, CM, and ZS conducted experiments and data acquisition. ZZ wrote the manuscript closely with all authors. XK and CM modified the manuscript. All the authors were involved in many discussion and revised the manuscript.

### Conflict of interest statement

The authors declare that the research was conducted in the absence of any commercial or financial relationships that could be construed as a potential conflict of interest.
