# Supplementary material for: The Amino Acid Metabolic and Carbohydrate Metabolic Pathway Play Important Roles during Salt-Stress Response in Tomato
Source: Front Plant Sci. 2017 Jul 17;8:1231. doi: 10.3389/fpls.2017.01231 (PMC5511834; doi:10.3389/fpls.2017.01231)
Supplement: Supplementary file 1 [file Presentation1.pdf]

SUPPLEMENTARY INFORMATION

RNA-seq data has been deposited in the sequence read archive (SRA) and the accession number is SRR5585697. Table S1, Primers of related genes for qRT-PCR. Table S2, The information of related regulatory genes in amino acid metabolic pathway. Table S3, The information of related regulatory genes in carbohydrate metabolic pathway.

Table S1. Primers of related genes for qRT-PCR.

Type	Gene	Sequence of primers（F-Primer 5'-3'）	Sequence of primers（R-Primer 5'-3'）
Pro1	Solyc05g051250.2.1	ATACAGAGAAGGCAGGGAA	TCAAGCAAACATAGCAAGAC
Pro2	Solyc01g091170.2.1	TCCAAAAGAAGTAGTCAAAAGAG	TCCAAGTCCACGAACAAG
Pro3	Solyc09g075070.2.1	TGACGCATACCATAAATACAA	CGACCACGAAATAGAAAATC
Pro4	Solyc06g060070.2.1	CCCTCCAAAACTCCTCAA	TTCATCCATTACTTCCATCAC
Pro5	Solyc07g026650.2.1	CGAGTGATGGCTGAAAAG	GAGGTGACAGGGATACAAGA
Pro6	Solyc12g011300.1.1	AAGAACAAGAGCAAGCAATC	TCATAGGCAAGAAACCAACT
Pro7	Solyc09g008670.2.1	CAGAGGGTGTTTTCATTCA	TTTGTGGGGTGGTTGTAG
Pro8	Solyc01g067860.2.1	CGGAAGAAAAGATGGGAA	CCTGATAACGCAACGAGA
Carboh1	Solyc06g062430.2.1	CTGAAGCCATTAGGAAAGAC	AACAGCCCATTGAGGAAG
Carboh2	Solyc07g055840.2.1	AGCACCCTATCTGTCTTTCA	TATTTGTTTATCCCTCACTTGTT
Carboh3	Solyc05g051250.2.1	ATACAGAGAAGGCAGGGAA	TCAAGCAAACATAGCAAGAC
Carboh4	Solyc01g094380.2.1	CTTCTTTCTGCTCGTAGTTTCT	TTGAGGTTGGATTGTTGG
Carboh5	Solyc01g110360.2.1	GGTCTTGATGGACTTGCC	ATGCTGCTTCCTTGACAG
Carboh6	Solyc09g075330.2.1	GCTTTGTTGATGGATGGA	ACTGCTTGATGCTTGATTG
Carboh7	Solyc10g083290.1.1	GAGAGTATTGGTTGGGAGTG	TGAGTGGAGTGGGTGTTT

Table S2. The information of related regulatory genes in amino acid metabolic pathway.

Gene ID	Gene description	Gene length
Solyc05g051250.2.1	glutamine synthetase-like	3317
Solyc10g078550.1.1	glutamate dehydrogenase	3043
Solyc01g091170.2.1	arginase 2	2219
Solyc02g089610.1.1	S-adenosylmethionine decarboxylase 2	1089
Solyc03g031730.2.1	beta-glucosidase 46-like	11145
Solyc09g075070.2.1	beta-glucosidase 11-like	5162
Solyc12g040640.1.1	beta-glucosidase 44-like	5209
Solyc06g060070.2.1	1-aminocyclopropane-1-carboxylate oxidase 5	2777
Solyc07g026650.2.1	1-aminocyclopropane-1-carboxylate oxidase	1429

Solyc08g081550.2.1	1-aminocyclopropane-1-carboxylate synthase-like	2650
Solyc09g011500.2.1	probable glutathione S-transferase	1584
Solyc09g011520.2.1	probable glutathione S-transferase	1118
Solyc12g011300.1.1	probable glutathione S-transferase	1128
Solyc09g008670.2.1	threonine dehydratase biosynthetic, chloroplastic	4104
Solyc01g067860.2.1	peroxidase 24	1599
Solyc03g080150.2.1	peroxidase 5	1595
Solyc07g017880.2.1	peroxidase 16	2057

Table S3. The information of related regulatory genes in carbohydrate metabolic pathway.

Gene ID	Gene description	Gene length
Solyc06g062430.2.1	inositol oxygenase 1	3421
Solyc09g007270.2.1	L-ascorbate peroxidase 2, cytosolic	4126
Solyc07g055840.2.1	citrate synthase 3, peroxisomal-like	8374
Solyc05g051250.2.1	glutamine synthetase-like	3317
Solyc10g007600.2.1	peroxisomal (S)-2-hydroxy-acid oxidase GLO1	4517
Solyc01g094380.2.1	O-glucosyltransferase rumi-like	4665
Solyc01g110360.2.1	fructose-bisphosphate aldolase 1, chloroplastic	2650
Solyc01g091050.2.1	pectinesterase 2	2917
Solyc02g072150.2.1	probable alpha,alpha-trehalose-phosphate synthase7	4080
Solyc03g031730.2.1	beta-glucosidase 46-like	11145
Solyc04g072920.2.1	probable trehalose-phosphate phosphatase J	2196
Solyc07g042520.2.1	sucrose synthase-like	3783
Solyc07g063880.2.1	putative beta-glucosidase 41	3978
Solyc07g064180.2.1	pectin esterase	3258
Solyc08g007130.2.1	beta-amylase 3, chloroplastic-like	3539
Solyc09g075070.2.1	beta-glucosidase 11-like	5162
Solyc09g075330.2.1	probable pectinesterase/pectinesterase inhibitor 7	2001
Solyc10g083290.1.1	acid invertase	5387
Solyc12g040640.1.1	beta-glucosidase 44-like	5209
